# *In Vivo* Activation and Pro-Fibrotic Function of NF-κB in Fibroblastic Cells During Pulmonary Inflammation and Fibrosis Induced by Carbon Nanotubes

**DOI:** 10.3389/fphar.2019.01140

**Published:** 2019-10-02

**Authors:** Jie Dong, Qiang Ma

**Affiliations:** Receptor Biology Laboratory, Toxicology and Molecular Biology Branch, Health Effects Laboratory Division, National Institute for Occupational Safety and Health, Centers for Disease Control and Prevention, Morgantown, WV, United States

**Keywords:** inflammation, fibrosis, NF-κB, carbon nanotube, fibroblast, myofibroblast, TIMP1, OPN

## Abstract

Exposure to insoluble particles in the lung elicits inflammatory responses that eliminate deposited particulates and repair damaged tissue. Overzealous or prolonged responses lead to chronic conditions, such as fibrosis and malignancy, which are frequently progressive and refractory to drug therapy leading to high rates of disability and mortality. The molecular events underlying the progression of lung inflammation to chronic pathology, in particular, the conversion to fibrosis, remain poorly understood. Fibrogenic multi-walled carbon nanotubes (MWCNTs) have been shown to stimulate prominent acute inflammation that evolves into chronic lesions characterized by chronic inflammation, interstitial fibrosis, and granulomas in mouse lungs. In this communication, we examined the *in vivo* activation of nuclear factor-κB (NF-κB) signaling in fibroblastic cells during the inflammatory and fibrotic progression induced by MWCNTs. Wild-type C57BL/6J male mice were exposed to two fibrogenic MWCNTs (Mitsui XNRI MWNT-7 and long MWCNTs) by pharyngeal aspiration. Both MWCNTs strongly stimulated the nuclear translocation of NF-κB p65 in lung fibroblasts and myofibroblasts during the acute and chronic responses. Phosphorylated NF-κB p65 at serine 276, a marker of NF-κB activation, was markedly induced by MWCNTs in the nucleus of fibroblastic cells. Moreover, two NF-κB-regulated genes encoding pro-fibrotic mediators, tissue inhibitor of metalloproteinase 1 (TIMP1), and osteopontin (OPN), respectively, were significantly induced in lung fibroblasts and myofibroblasts. These results demonstrate that NF-κB is activated to mediate transactivation of pro-fibrotic genes in fibroblastic cells during pulmonary acute and chronic responses to CNTs, providing a mechanistic framework for analyzing gene regulation in pulmonary fibrotic progression through NF-κB signaling.

## Introduction

Humans are constantly exposed to micro and nano size particulates, such as mineral dusts, microbial and organic bodies, and nanomaterials. Upon inhalation, insoluble particles deposit in the airway and the alveolar space and elicit acute inflammatory responses in the lung, including infiltration of granulocytes, activation of macrophages, and production and secretion of pro-inflammatory mediators. These inflammatory events help to clear deposited particles, repair damaged lung tissue, and restore pulmonary homeostasis. Under pathological conditions, pulmonary inflammation converts to chronic lesions that promote the development of fibrosis and cancer, exemplified by pneumoconiosis, lung cancer, and mesothelioma. These chronic disease conditions are frequently progressive and resistant to treatment, resulting in high mortality and medical burden. Understanding the pathogenesis of chronic lung disease associated with particulate exposure at molecular and cellular levels has been difficult due to the lack of appropriate animal models.

Carbon nanotubes (CNTs) are long and hollow cylindrical nanostructures made of one-atom-thick graphene sheets. Owing to their unique properties, CNTs have been developed with a broad range of applications. Production of CNTs and CNT-containing materials has been increased substantially in recent two decades (De Volder et al., 2013; Zhang et al., 2013). On the other hand, the nano-scaled size, fiber-like shape, and high biopersistence of CNTs present certain risk to the health of exposed populations. In laboratory animals, CNTs elicit a number of adverse effects, among which pulmonary inflammation and fibrosis have emerged as predominant outcomes (Dong and Ma, 2015; Dong and Ma, 2016; Vietti et al., 2016; Dong and Ma, 2018; Duke and Bonner, 2018). CNT-induced lung fibrosis is characterized by elevated production and secretion of pro-fibrotic mediators, increased numbers of fibrosis effector cells including fibroblasts and myofibroblasts, excessive deposition and accumulation of the extracellular matrix (ECM), and replacement of lung tissues with scar formed from fibrotic foci and granulomas. Consistent with laboratory findings, field studies identified elevated levels of pro-fibrotic factors and fibrosis markers in the body fluids of workers exposed to CNTs at workplace (Liou et al., 2015; Fatkhutdinova et al., 2016). At the pathological level, CNT-induced lung fibrosis shares certain features with some human fibrotic lung diseases, such as idiopathic pulmonary fibrosis (IPF) and pneumoconiosis. Elucidating the mechanism underlying CNT-induced lung fibrosis is a prerequisite for the assessment and intervention of CNT lung pathology and would facilitate the overall understanding of human fibrotic lung diseases.

Nuclear factor-κB (NF-κB) is a key transcription factor in the regulation of inflammation, immune response, and tumorigenesis. In these scenarios, NF-κB controls the transcription of target genes encoding critical regulators or effectors of host responses to intracellular and extracellular stresses (Pahl, 1999; Ghosh and Karin, 2002; Lawrence, 2009). Activation of NF-κB and the spectrum of its target genes being activated are often inducer-, cell type-, and context-dependent. Among the most well-studied functions of NF-κB, NF-κB mediates the up-regulation of pro-inflammatory cytokines, such as TNF-α, IL-1β, and IL-6, which are largely produced by classically activated (M1) macrophages during the acute inflammatory response to pathogens, environmental stimuli, and inflammatory cues from the tissue microenvironment. Aberrant NF-κB functions have been implicated in the development of chronic inflammation and malignancy (Basseres and Baldwin, 2006; Ben-Neriah and Karin, 2011; Perkins, 2012). In lung diseases, elevated or prolonged NF-κB activation is detected in the lungs of patients with asthma, chronic obstructive pulmonary disease (COPD), and silicosis (Wright and Christman, 2003; Di Giuseppe et al., 2009; Edwards et al., 2009).

NF-κB was activated by single-walled CNTs (SWCNTs) in cultured cells, including human HaCaT keratinocytes (Manna et al., 2005), human mesothelial cells (Pacurari et al., 2008), mouse epidermal JB6 P+ cells (Murray et al., 2009), rat aortic endothelial cells (RAEC) (Zhiqing et al., 2010), and mouse RAW264.7 macrophages (He et al., 2012). Treatment with multi-walled CNTs (MWCNTs) led to NF-κB activation and increased IL-8 mRNA expression, which was suppressed by pre-treatment with an NF-κB inhibitor, in human alveolar epithelial A549 cells (Ye et al., 2009). In RAW264.7 macrophages, MWCNTs induced the activation of NF-κB signaling and increased the secretion of pro-inflammatory cytokines and chemokines, such as TNF-α, IL-1β, IL-6, IL-10, and MCP-1 (He et al., 2011). MWCNTs also induced phosphorylation of IκBα and the nuclear accumulation of NF-κB in rat lung epithelial cells (Ravichandran et al., 2010).

Despite *in vitro* findings, studies on the effects of CNTs on NF-κB activation and function in intact animals have been scarce. As such, the role and mode of action of NF-κB in CNT pathology *in vivo* remain largely unexplored. Pathway analysis of Affymetrix microarray data from mouse lungs exposed to SWCNTs through intratracheal instillation suggested that NF-κB-related inflammatory responses and downstream signals affecting tissue remodeling play a role in SWCNT-induced pathologic effects (Chou et al., 2008; Hsieh et al., 2012). Treatment with an NF-κB inhibitor attenuated SWCNT-induced airway hyperreactivity and chronic airway inflammation in mice, although direct evidence on the activation of NF-κB by SWCNTs was not provided (Hsieh et al., 2012). These *in vitro* and *in vivo* findings discussed above are consistent with the notion that NF-κB is required for the production of pro-inflammatory cytokines from activated macrophages during acute inflammation in the lung. The role and mode of action of NF-κB in other types of cells, such as lung fibroblasts and myofibroblasts that are major effector cells for fibrosis development in the lung, remain unclear but could be critical in pulmonary chronic progression to fibrosis. Therefore, *in vivo* study on how MWCNTs influence the NF-κB pathway in different types of cells during chronic progression to fibrosis, and the functional impact of NF-κB activation in these cells on MWCNT-induced lung fibrosis is much needed.

In the current study, we examined the activation and signaling of NF-κB and its transcriptional activity on pro-fibrotic target genes for fibrosis development in mouse lungs exposed to MWCNTs with focus on fibroblasts and myofibroblasts, as these cells play a major role in matrix production and scarring during fibrosis. The findings reveal that MWCNTs induce the activation of NF-κB in the lung during both the acute and chronic responses. Specifically, NF-κB is activated by MWCNTs in fibroblasts and myofibroblasts in mouse lungs and promotes the expression of tissue inhibitor of metalloproteinase 1 (TIMP1) and osteopontin (OPN), which are pro-fibrotic factors in MWCNT-induced lung fibrosis. Our study provides a framework for *in vivo* analysis of NF-κB signaling and function in fibroblastic cells for CNT-induced inflammation and fibrosis in mammalian lungs.

## Materials and Methods

### Multi-Walled Carbon Nanotubes

MWNT-7 MWCNTs were obtained from Mitsui & Company (XNRI MWNT-7, lot #05072001K28, Tokyo, Japan). Characterization of these MWCNTs has been conducted and reported previously (Porter et al., 2010; Dong and Ma, 2017a). Briefly, the MWNT-7 MWCNTs have a diameter of 49 ± 13.4 nm with normal distribution and a length of 3.86 ± 1.94 μm with log-normal distribution. Trace element contaminations were 0.78% for all metals, 0.41% for sodium, and 0.32% for iron. Long MWCNTs were obtained from Cheap Tubes Inc. (Cambridgeport, VT, USA) and have an outer diameter of 30–50 nm and a length of 10–20 μm. Previous studies have shown that, in animal lungs, both MWNT-7 MWCNTs and long MWCNTs induce remarkable fibrotic responses (Dong and Ma, 2015; Vietti et al., 2016; Duke and Bonner, 2018). A dispersion medium (DM), containing 0.6 mg/ml mouse serum albumin (Sigma–Aldrich, St. Louis, MO, USA) and 0.01 mg/ml 1,2-dipalmitoyl-sn-glycerol-3-phosphocholine (Sigma–Aldrich) in Ca^2+^- and Mg^2+^-free PBS, pH7.4, was used to disperse MWCNTs and as the vehicle control for treatment. DM and MWCNT suspensions were freshly prepared before use following a procedure described previously (Porter et al., 2010).

### Animals and Treatment

Eight- to 10-week-old male C57BL/6J mice were purchased from The Jackson Laboratory (Bar Harbor, ME, USA) and maintained in an accredited, specific pathogen-free, and environmentally controlled facility at the National Institute for Occupational Safety and Health. All experiments involving animals were approved by the Institutional Animal Care and Use Committee.

A single dose of 50 µl DM or 40 μg MWCNTs in 50 µl DM was administered by pharyngeal aspiration to deliver MWCNTs into mouse lungs with an even distribution (Rao et al., 2003; Porter et al., 2010). The selected dose of MWCNTs at 40 μg per mouse is based on previous dose–response studies. At this dose, the MWCNTs induced apparent acute and chronic fibrosis in mouse lungs. Meanwhile, this dose is relevant to human exposure at workplace as described previously (Dong et al., 2015; Dong and Ma, 2017a). There was no significant difference in the initial body weights among treatment groups. Mice were monitored for general health conditions daily after treatment. No apparent effects on the overall health from the treatment were observed during exposure.

### Immunofluorescence

Cryostat sections from frozen left lung lobe (7 µm) were fixed with 4% paraformaldehyde and used for immunofluorescence as described previously (Dong and Ma, 2016; Dong and Ma, 2017a; Dong and Ma, 2017b). Briefly, the slides were blocked for 1 h at room temperature, immunostained with primary antibodies at 4°C overnight, incubated with Alexa Fluor 488– or Alexa Fluor 594–conjugated secondary antibodies (Thermo Fisher Scientific, Waltham, MA, USA) for 1 h at room temperature in dark, and mounted with ProLong Diamond Antifade Mountant with DAPI (Thermo Fisher Scientific). Since mouse primary antibodies were applied, the blocking reagent and antibody diluent from the M.O.M. Immunodetection Kit (Vector Laboratories, Burlingame, CA, USA) were used to eliminate background staining. The primary antibodies used for immunofluorescence were anti-p65 (Santa Cruz, Dallas, TX, USA, sc-372, rabbit polyclonal), anti-Hsp47 (EMD Millipore, Billerica, MA, USA, 386023, mouse monoclonal), anti-α-SMA (Sigma–Aldrich, A2547, mouse monoclonal), anti-p-NF-κB p65 S276 (Santa Cruz, sc-101749, rabbit polyclonal), anti-TIMP1 (R&D systems, Minneapolis, MN, USA, AF980, goat polyclonal), and anti-OPN (R&D systems, AF808, goat polyclonal) antibodies. All primary antibodies were used at 1:200 dilution. Specificities of anti-TIMP1 and anti-OPN antibodies were confirmed using Alexa Fluor 488–conjugated secondary antibodies in the absence or presence of normal goat IgG (Santa Cruz, sc-2028) as controls under the same conditions as for anti-TIMP1 and anti-OPN antibodies (**Figure S9**). All studies were performed 2–3 times independently to confirm the results. Images were taken with a Zeiss LSM 780 confocal microscope (Carl Zeiss Microscopy, Jena, Germany). The numbers of positively stained cells on microscopic images (image area: 1.864x10^4^ μm^2^) were determined using the ImageJ program (*National Institutes of Health*, Bethesda, MD, USA). Numbers of cells per unit area (2x10^4^ μm^2^) were presented as the mean ± SD (*n* = 4).

### Statistical Analysis

The statistical analysis of differences between experimental groups was performed with one-way ANOVA followed by between group comparisons using standard procedures (https://astatsa.com/OneWay_Anova_with_TukeyHSD/). Representative data were presented as the mean ± SD. A *p* value of less than 0.05 was considered statistically significant. *, *p* < 0.05; **, *p* < 0.01; and ***, *p* < 0.001.

## Results

### Activation of NF-κB in Fibroblasts and Myofibroblasts in MWCNT-Exposed Lungs

Pulmonary exposure to MWCNTs induces an acute fibrotic response in the lung that becomes evident on day 3 and reaches a peak on day 7. The acute response is largely diminished during the second week post-exposure, followed by a chronic response characterized by interstitial fibrosis and granuloma formation. The chronic fibrotic lesions become prominent on day 28 post-exposure (Dong et al., 2015; Dong and Ma, 2017a). To examine the activation of NF-κB signaling during fibrosis development induced by MWCNTs, we chose day 3 and day 7 post-exposure for the acute response and day 28 for the chronic response to analyze. Two fibrogenic MWCNTs, i.e., the short and rigid MWNT-7 MWCNTs and the long MWCNTs, were used to stimulate lung fibrosis. As fibroblasts and myofibroblasts are the major effector cells in organ fibrosis and are highly enriched in the fibrotic foci in CNT-exposed lungs (Dong and Ma, 2016; Dong and Ma, 2017a; Dong and Ma, 2017b), analyses of NF-κB activation and function were focused in these fibroblastic cells.

**Figure 1:**
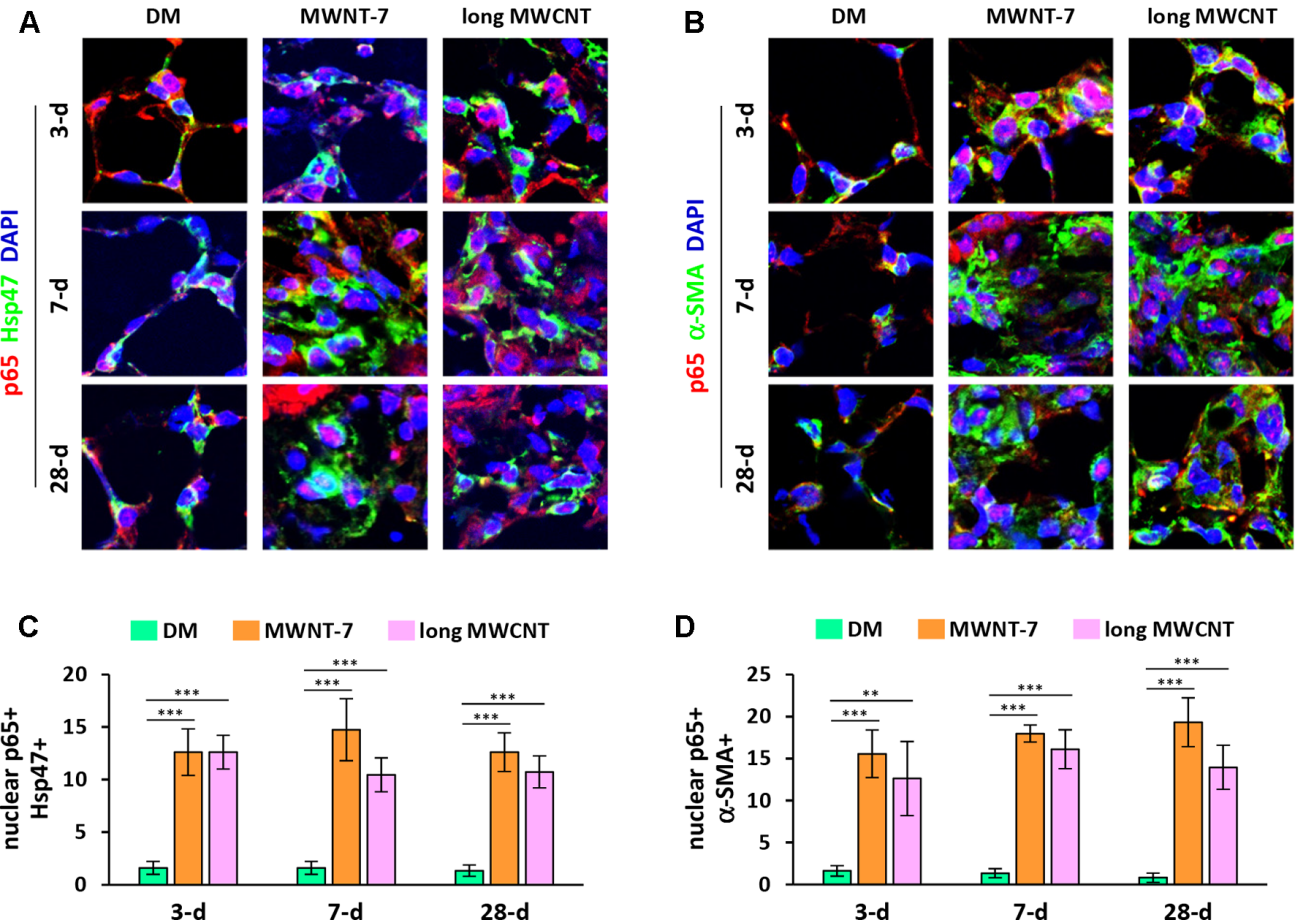
Induction of nuclear translocation of p65 in fibroblastic cells by MWCNTs in the lung. **(A)** Nuclear p65 in fibroblasts examined by double immunofluorescence staining of p65 (red) and Hsp47 (green), with DAPI nuclear staining (blue). The full images with scale bar are shown in **Figure S1**. **(B)** Nuclear p65 in myofibroblasts examined by double immunofluorescence staining of p65 (red) and α-SMA (green), with DAPI nuclear staining (blue). The full images with scale bar are shown in **Figure S2**. In **(A)** and **(B)**, pink color generated from the overlap of red and blue indicates nuclear p65. **(C)** Number of nuclear p65+ Hsp47+ cells per unit area (2x10^4^ µm^2^) is presented as mean ± SD (*n* = 4). **(D)** Number of nuclear p65+ α-SMA+ cells per unit area (2x10^4^ µm^2^) is presented as mean ± SD (*n* = 4).

Activation of the canonical NF-κB signaling pathway involves the release of an NF-κB heterodimer, p50:p65, from its inhibitor IκB and the translocation of the p50:p65 dimer from the cytoplasm to the nucleus. Upon entering the nucleus, the p50:p65 heterodimer binds to κB-binding sites on DNA and activates transcription of target genes. Therefore, nuclear translocation of p65 protein is generally considered as a hallmark for NF-κB activation. We first examined the localization of p65 in MWCNT-exposed lungs with double immunofluorescence staining. DAPI was used for nuclear staining. Hsp47 (heat shock protein 47) was used as the marker for fibroblasts, whereas α-SMA (α-smooth muscle actin) for myofibroblasts. Both MWNT-7 MWCNTs and long MWCNTs increased the numbers of fibroblasts and myofibroblasts in fibrotic foci during fibrosis development as expected. Both MWCNTs evidently induced the translocation of p65 to the nucleus in fibroblasts during both the acute and chronic phase responses to MWCNT exposure in the lung, demonstrated by the pink color generated from the overlap of the red color (p65 staining) and the blue color (nuclear staining) on images (**Figures 1A** and **S1**). Similarly, MWNT-7 MWCNTs and long MWCNTs markedly induced the presence of p65 in the nucleus in myofibroblasts at all the time points studied (**Figures 1B** and **S2**). Induction of nuclear p65+ fibroblasts (**Figure 1C**) or nuclear p65+ myofibroblasts (**Figure 1D**) by MWNT-7 or long MWCNTs was statistically significant at all the time points examined, in comparison with DM control at the same time point. The increased numbers of fibroblasts and myofibroblasts that were positive of p65 in the nucleus indicate activation of NF-κB by MWCNTs in these cells and suggest a role of NF-κB activation in MWCNT-elicited fibrosis development.

**Figure 2:**
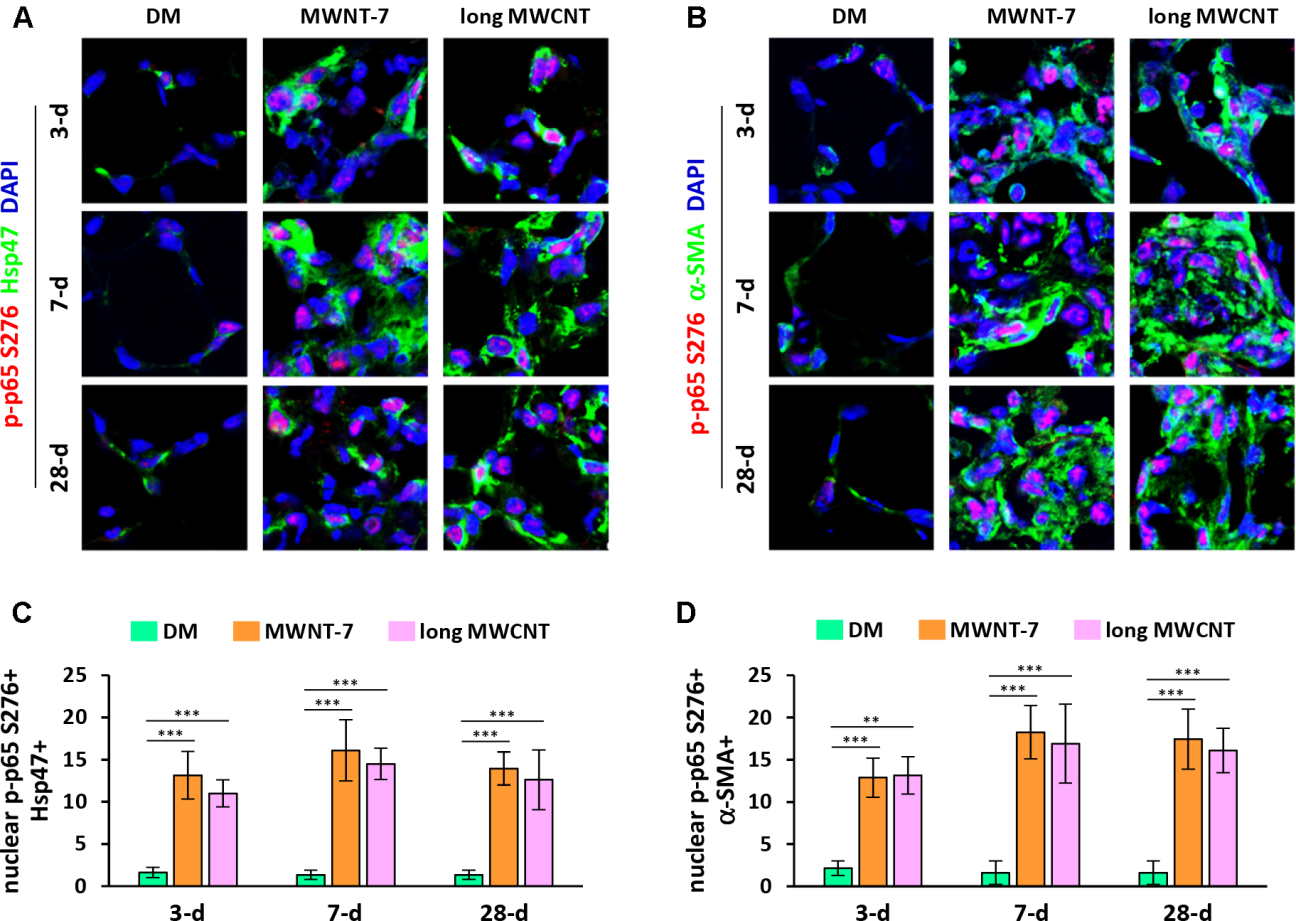
Induction of phosphorylation of p65 at S276 in fibroblastic cells by MWCNTs in the lung. **(A)** Nuclear p-p65 S276 in fibroblasts examined by double immunofluorescence staining of p-p65 S276 (red) and Hsp47 (green), with DAPI nuclear staining (blue). The full images with scale bar are shown in **Figure S3**. **(B)** Nuclear p-p65 S276 in myofibroblasts examined by double immunofluorescence staining of p-p65 S276 (red) and α-SMA (green), with DAPI nuclear staining (blue). The full images with scale bar are shown in **Figure S4**. In **(A)** and **(B)**, pink color generated from the overlap of red and blue indicates nuclear p-p65 S276. **(C)** Number of nuclear p-p65 S276+ Hsp47+ cells per unit area (2x10^4^ µm^2^) is presented as mean ± SD (*n* = 4). **(D)** Number of nuclear p-p65 S276+ α-SMA+ cells per unit area (2x10^4^ µm^2^) is presented as mean ± SD (*n* = 4).

In addition to nuclear translocation, posttranslational modifications of p65 play important roles in NF-κB activation and function (Ghosh and Karin, 2002; Christian et al., 2016). Phosphorylation of p65 at serine 276 (S276) is a critical step in NF-κB activation and NF-κB-dependent gene expression. Double immunofluorescence staining on lung tissue sections demonstrated markedly increased levels of phosphorylated p65 at S276 in the nucleus in both fibroblasts (**Figures 2A** and **S3**) and myofibroblasts (**Figures 2B** and **S4**) induced by MWNT-7 or long MWCNTs, which was evidenced by the pink color generated from the overlap of p-p65 S276 staining (red) and nuclear staining (blue) and was observed at all the time points examined. Quantification reveals that the numbers of nuclear p-p65 S276+ fibroblasts (**Figure 2C**) and nuclear p-p65 S276+ myofibroblasts (**Figure 2D**) were significantly increased by MWNT-7 or long MWCNTs at all the time points detected, in comparison with DM control at the same time point. The majority of p-p65 S276 staining was found to locate in the nucleus in fibroblasts and myofibroblasts in MWCNT-exposed lungs (**Figures 2A, B**), consistent with the activation of NF-κB in fibroblasts and myofibroblasts during MWCNT-induced lung fibrosis development.

### Induction of NF-κB-Regulated Pro-Fibrotic Mediators in Fibroblasts and Myofibroblasts by MWCNTs in the Lung

NF-κB modulates cellular functions by transactivating target genes. We have previously shown that two NF-κB-regulated genes, Timp1 and Opn, are significantly induced by MWCNTs in the lung during fibrosis development, and both TIMP1 and OPN play critical roles in promoting MWCNT-induced lung fibrosis (Dong and Ma, 2017a; Dong and Ma, 2017b). We therefore examined if the expression of TIMP1 and OPN was elevated in fibroblasts and myofibroblasts in accordance with NF-κB activation in MWCNT-exposed lungs.

**Figure 3:**
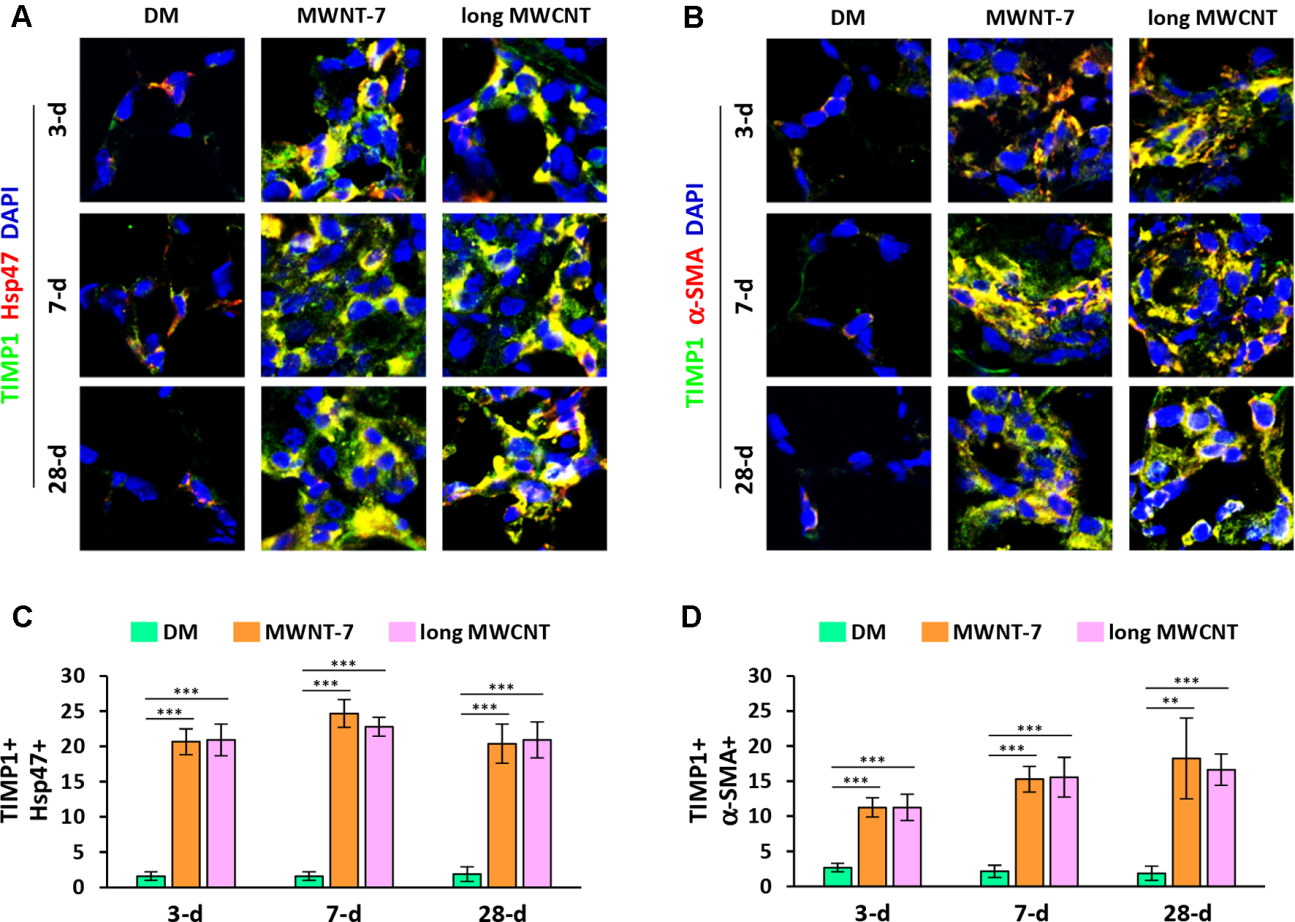
Elevated expression of TIMP1 in fibroblastic cells by MWCNTs in the lung. **(A)** TIMP1 in fibroblasts examined by double immunofluorescence staining of TIMP1 (green) and Hsp47 (red), with DAPI nuclear staining (blue). The full images with scale bar are shown in **Figure S5**. **(B)** TIMP1 in myofibroblasts examined by double immunofluorescence staining of TIMP1 (green) and α-SMA (red), with DAPI nuclear staining (blue). The full images with scale bar are shown in **Figure S6**. In **(A)** and **(B)**, yellow color is generated from the overlap of green and red. **(C)** Number of TIMP1+ Hsp47+ cells per unit area (2x10^4^ µm^2^) is presented as mean ± SD (*n* = 4). **(D)** Number of TIMP1+ α-SMA+ cells per unit area (2x10^4^ µm^2^) is presented as mean ± SD (*n* = 4).

Using double immunofluorescence staining on lung tissue sections, both MWNT-7 and long MWCNTs were shown to increase the level of TIMP1 in fibroblasts during the acute and chronic responses evidently, indicated by the yellow color generated from the overlap of green color (TIMP1 staining) and red color (Hsp47 staining) on images (**Figures 3A** and **S5**). Similarly, the level of TIMP1 was markedly increased in myofibroblasts by MWCNTs at all the time points examined, presented by the yellow color generated from the overlap of green color (TIMP1 staining) and red color (α-SMA staining) on images (**Figures 3B** and **S6**). Induction of TIMP1+ fibroblasts (**Figure 3C**) or TIMP1+ myofibroblasts (**Figure 3D**) by MWNT-7 or long MWCNTs was statistically significant on days 3, 7, and 28 post-exposure, compared with DM control at the same time point. This study therefore reveals that NF-κB up-regulates the expression of TIMP1, a pro-fibrotic mediator, in fibroblasts and myofibroblasts in response to MWCNT exposure *in vivo*, providing an evidence for the implication of NF-κB in MWCNT-initiated lung fibrosis development.

**Figure 4:**
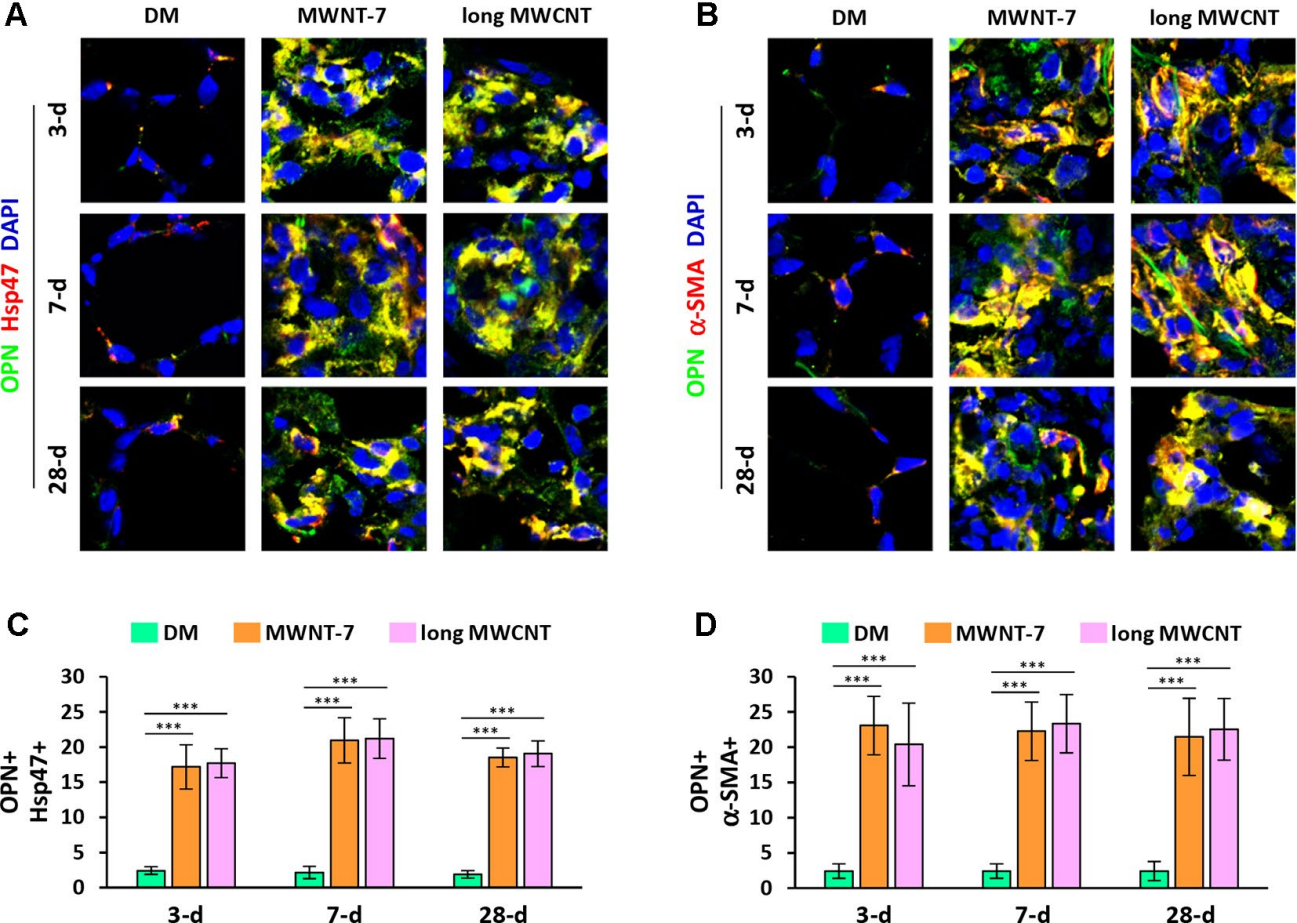
Increased expression of OPN in fibroblastic cells by MWCNTs in the lung. **(A)** OPN in fibroblasts examined by double immunofluorescence staining of OPN (green) and Hsp47 (red), with DAPI nuclear staining (blue). The full images with scale bar are shown in **Figure S7**. **(B)** OPN in myofibroblasts examined by double immunofluorescence staining of OPN (green) and α-SMA (red), with DAPI nuclear staining (blue). The full images with scale bar are shown in **Figure S8**. In **(A)** and **(B)**, yellow color is generated from the overlap of green and red. **(C)** The number of OPN+ Hsp47+ cells per unit area (2x10^4^ µm^2^) is presented as mean ± SD (*n* = 4). **(D)** The number of OPN+ α-SMA+ cells per unit area (2x10^4^ µm^2^) is presented as mean ± SD (*n* = 4).

Double immunofluorescence staining on lung tissue sections also uncovered that the expression of OPN was apparently induced by both MWNT-7 and long MWCNTs in fibroblasts during fibrosis development, indicated by the yellow color generated from the overlap of green color (OPN staining) and red color (Hsp47 staining) on images (**Figures 4A** and **S7**). Likewise, the level of OPN was obviously elevated in myofibroblasts by MWCNTs during the acute and chronic responses, shown by the yellow color generated from the overlap of green color (OPN staining) and red color (α-SMA staining) on images (**Figures 4B** and **S8**). Moreover, induction of OPN+ fibroblasts (**Figure 4C**) or OPN+ myofibroblasts (**Figure 4D**) by MWNT-7 or long MWCNTs was statistically significant at all the time points detected, compared with DM control at the same time point. This study therefore demonstrates that the NF-κB-regulated pro-fibrotic factor OPN is highly induced by MWCNTs in fibroblastic cells in the lung, providing another molecular mediator that links NF-κB activation to MWCNT-induced lung fibrosis.

## Discussion

Several studies reported that NF-κB is activated by CNTs in cultured cells (Manna et al., 2005; Pacurari et al., 2008; Murray et al., 2009; Ravichandran et al., 2010; He et al., 2011; He et al., 2012). Whether NF-κB is activated *in vivo* and plays a role in CNT-induced lung pathology remains unaddressed. Given the critical roles of NF-κB in the development of chronic inflammation, organ fibrosis, and tumorigenesis in human disease, we examined the activation of NF-κB in the lung during MWCNT-induced lung fibrosis, one of the most concerned pathologic outcomes of MWCNT exposure. The fibroblastic cells, i.e., fibroblasts and myofibroblasts, were specifically studied, as these are the predominant cells enriched in fibrotic foci and the major effector cells for fibrosis development (Dong and Ma, 2016). For example, we have previously shown that exposure to MWNT-7 increased the numbers of fibroblasts and myofibroblasts in fibrotic lung tissues, which were substantially and significantly higher than those in control lungs (Dong and Ma, 2017a). Notwithstanding, the mechanism by which fibroblastic cells mediate fibrosis progression and how the cells are regulated during fibrotic disease development at molecular levels remain poorly understood, which hinders drug targeting against fibrosis diseases.

Two lines of evidence obtained from this study revealed the activation of NF-κB by MWCNTs in fibroblastic cells in the lung. First, we showed the nuclear localization of NF-κB subunit p65, which is a necessary step in the activation of the canonical pathway of NF-κB signaling, supporting that NF-κB activation was induced by two types of MWCNTs in fibroblasts and myofibroblasts during both the acute phase and the chronic phase of fibrosis development (**Figures 1**, **S1**, and **S2**). Second, we revealed that phosphorylated p65 at S276 was evidently induced and located in the nucleus in fibroblasts and myofibroblasts in MWCNT-exposed lungs (**Figures 2**, **S3**, and **S4**). Phosphorylation of p65 at S276 induced by stimuli through protein kinase A (PKA) has been demonstrated to be critical for NF-κB transcriptional activity downstream of IκB degradation (Chen and Greene, 2004). In resting cells, PKA and cytosolic p50:p65:IκB form a complex. Upon stimulation, following IκB degradation, PKA phosphorylates p65 at S276, and then the p50:p65 heterodimer translocates to the nucleus and binds to κB-binding sites on DNA. Phosphorylation of p65 at S276 promotes the interaction of p65 with the transcription coactivators CBP (CREB-binding protein) and p300, which are essential for gene transcription (Zhong et al., 1998). Therefore, phosphorylated p65 at S276 is another hallmark of NF-κB activation and determines transactivation by NF-κB. Together, these findings confirmed the activation of NF-κB by MWCNTs *in vivo* during fibrosis development.

As the primary mode of action of NF-κB involves the regulation of transcription of target genes, we analyzed the expression of NF-κB target genes associated with fibrogenic responses. We have previously found that TIMP1 serves as a pro-fibrotic factor and promotes MWCNT-induced lung fibrosis (Dong and Ma, 2017b). TIMP1 was rapidly and highly induced by MWNT-7 in the lung in a time- and dose-dependent manner, with high levels detected in lung tissue, the bronchoalveolar lavage (BAL) fluid, and the serum. Knockout of Timp1 in mice caused a significant attenuation in fibrotic focus formation, collagen fiber deposition, fibroblast accumulation, and myofibroblast differentiation in the lung exposed to MWCNTs, compared with wild-type. Notably, MWCNTs significantly increased the expression of cell proliferation markers Ki-67 and PCNA (proliferating cell nuclear antigen) and a panel of cell cycle–controlling genes in the lung in a TIMP1-dependent manner. At the molecular level, MWCNTs elicited a significant induction of CD63 and integrin β1 in lung fibroblasts and the formation of a TIMP1/CD63/integrin β1 complex on the surface of fibroblasts, which triggers the phosphorylation and activation of the extracellular signal-regulated kinase (Erk) 1/2. Overall, this study supports that the pulmonary fibrotic response to MWCNTs involves, at least in part, the induction of TIMP1, which promotes the fibroblast activation and proliferation through activation of the TIMP1/CD63/integrin β1 axis and ERK signaling. How TIMP1 is induced by MWCNTs in the lung was unaddressed previously. On the other hand, Timp1 gene expression was found to be up-regulated by NF-κB in multiple studies, demonstrating Timp1 is a target gene of NF-κB (Wilczynska et al., 2006; Xia et al., 2012; Gong et al., 2015). Activation of NF-κB by MWCNTs serves as a potential mechanism for induction of TIMP1 in mouse lungs. Therefore, we studied the expression of TIMP1 in fibroblasts and myofibroblasts, in which NF-κB was activated, in response to MWCNT exposure. Indeed, the findings uncover that the level of TIMP1 was markedly increased in fibroblasts and myofibroblasts in MWCNT-exposed lungs, consistent with the activation of NF-κB (**Figures 3**, **S5**, and **S6**). This result reveals a link between NF-κB activation and MWCNT-induced lung fibrosis through NF-κB-regulated up-regulation of pro-fibrotic factor TIMP1.

Opn is another NF-κB-regulated gene, as demonstrated in multiple studies (Samant et al., 2007; Irita et al., 2008; Zhao et al., 2011; Guarneri et al., 2017). OPN was highly and persistently induced by MWCNTs or SWCNTs in both the acute and chronic responses in lung tissue and the BAL fluid in various studies (Huizar et al., 2011; Taylor et al., 2014; Fujita et al., 2015; Khaliullin et al., 2015; Malur et al., 2015; Thompson et al., 2015; Dong and Ma, 2017a). Comparison between wild-type and Opn knockout mice revealed that OPN is a potent pro-fibrotic factor in MWCNT-induced lung fibrosis (Dong and Ma, 2017a). OPN boosts MWCNT-induced lung fibrosis by promoting the formation of fibrotic foci and increasing the production of matrix proteins in the lung. At the cellular and molecular levels, OPN enhances TGF-β1 (transforming growth factor-β1) expression and activation, Smad-dependent TGF-β1 signaling activation, fibroblast accumulation, myofibroblast differentiation, and ECM production and deposition in MWCNT-exposed lungs. Using TGF-β1 neutralizing antibodies and a type I TGF-β receptor inhibitor, we showed that OPN promotes MWCNT-induced fibrotic response through activating TGF-β1 signaling and elevating ECM production in fibroblastic cells. Together, these findings reveal a pro-fibrotic activity of OPN in lung fibroblastic cells exposed to MWCNTs, through which OPN critically aggravates the pulmonary fibrotic response to MWCNT exposure. In the current study, we visualized that, in fibroblasts and myofibroblasts in MWCNT-exposed lungs, NF-κB was evidently activated, and the expression of its target gene Opn was highly induced, compared with DM-treated control lungs (**Figures 4**, **S7**, and **S8**). This study therefore reveals another link between NF-κB activation and MWCNT-induced lung fibrosis, which is bridged by the NF-κB-regulated, pro-fibrotic factor OPN.

In conclusion, we identified a molecular mechanism to underlie MWCNT-induced lung fibrosis development, which involves the activation of NF-κB signaling and the induced expression of its target genes encoding pro-fibrotic mediators TIMP1 and OPN, in fibroblasts and myofibroblasts *in vivo*. This mechanism is induced and activated by two types of MWCNTs in both the acute and chronic fibrotic responses, in mouse lungs. Therefore, this study suggests a role of NF-κB in fibrosis development through up-regulating the expression of pro-fibrotic genes. The findings provide a molecular insight into the mechanism of lung fibrosis induced by MWCNTs and provide a framework for further studies on NF-κB signaling and CNT-induced fibrosis. Such studies would reveal new targets for drug development and new strategies for therapy against pulmonary chronic inflammation and fibrosis in humans.

## Data Availability Statement

All datasets generated for this study are included in the manuscript/**Supplementary Files**.

## Ethics Statement

The animal study was reviewed and approved by the Institutional Animal Care and Use Committee at NIOSH/HELD.

## Author Contributions

JD designed and performed the experiments and prepared a manuscript. QM revised and finalized the article. Both authors read and approved the final manuscript.

## Funding

This study was funded to QM by the Health Effects Laboratory Division and the Nanotechnology Research Center at National Institute for Occupational Safety and Health, Centers for Disease Control and Prevention, USA (No. 8939050W).

## Disclaimer

The findings and conclusions in this report are those of the authors and do not necessarily represent the official position of the National Institute for Occupational Safety and Health, Centers for Disease Control and Prevention.

## Conflict of Interest

The authors declare that the research was conducted in the absence of any commercial or financial relationships that could be construed as a potential conflict of interest.
