# Supplementary material for: In Vivo Activation and Pro-Fibrotic Function of NF-κB in Fibroblastic Cells During Pulmonary Inflammation and Fibrosis Induced by Carbon Nanotubes
Source: Front Pharmacol. 2019 Oct 2;10:1140. doi: 10.3389/fphar.2019.01140 (PMC6783511; doi:10.3389/fphar.2019.01140)
Supplement: Supplementary file 1 [file DataSheet_1.pdf]

Supplementary figure legends

Fig. S1 Induction of nuclear translocation of p65 in fibroblasts by MWCNTs in the lung. Full microscopic pictures of the images presented in Fig. 1a, with the staining of p65 (red), Hsp47 (green), and DAPI nuclear staining (blue). Pink color generated from the overlap of red and blue indicates nuclear p65 (scale bar: 20 μm)

Fig. S2 Induction of nuclear translocation of p65 in myofibroblasts by MWCNTs in the lung. Full microscopic pictures of the images presented in Fig. 1b, with the staining of p65 (red), α-SMA (green), and DAPI nuclear staining (blue). Pink color generated from the overlap of red and blue indicates nuclear p65 (scale bar: 20 μm)

Fig. S3 Induction of phosphorylation of p65 at S276 in fibroblasts by MWCNTs in the lung. Full microscopic pictures of the images presented in Fig. 2a, with the staining of p-p65 S276 (red), Hsp47 (green), and DAPI nuclear staining (blue). Pink color generated from the overlap of red and blue indicates nuclear p-p65 S276 (scale bar: 20 μm)

Fig. S4 Induction of phosphorylation of p65 at S276 in myofibroblasts by MWCNTs in the lung. Full microscopic pictures of the images presented in Fig. 2b, with the staining of p-p65 S276 (red), α-SMA (green), and DAPI nuclear staining (blue). Pink color generated from the overlap of red and blue indicates nuclear p-p65 S276 (scale bar: 20 μm)

Fig. S5 Elevated expression of TIMP1 in fibroblasts by MWCNTs in the lung. Full microscopic pictures of the images presented in Fig. 3a, with the staining of TIMP1 (green), Hsp47 (red), and DAPI nuclear staining (blue). Yellow color is generated from the overlap of green and red (scale bar: 20 μm)

Fig. S6 Elevated expression of TIMP1 in myofibroblasts by MWCNTs in the lung. Full microscopic pictures of the images presented in Fig. 3b, with the staining of TIMP1 (green), α-SMA (red), and DAPI nuclear staining (blue). Yellow color is generated from the overlap of green and red (scale bar: 20 μm)

Fig. S7 Increased expression of OPN in fibroblasts by MWCNTs in the lung. Full microscopic pictures of the images presented in Fig. 4a, with the staining of OPN (green), Hsp47 (red), and DAPI nuclear staining (blue). Yellow color is generated from the overlap of green and red (scale bar: 20 μm)

Fig. S8 Increased expression of OPN in myofibroblasts by MWCNTs in the lung. Full microscopic pictures of the images presented in Fig. 4b, with the staining of OPN (green), α-SMA (red), and DAPI nuclear staining (blue). Yellow color is generated from the overlap of green and red (scale bar: 20 μm)

Fig. S9 Specificities of anti-TIMP1 and anti-OPN antibodies. Sections of lung tissues obtained on day 7 post-exposure to MWNT-7 were used. The specificities of anti-TIMP1 and anti-OPN antibodies were determined using Alexa Fluor 488-conjugated secondary antibodies in the absence or presence of normal goat IgG as controls, with DAPI nuclear staining (blue). The images were photographed under the same microscopic settings (scale bar: 20 μm)

Fig. S1

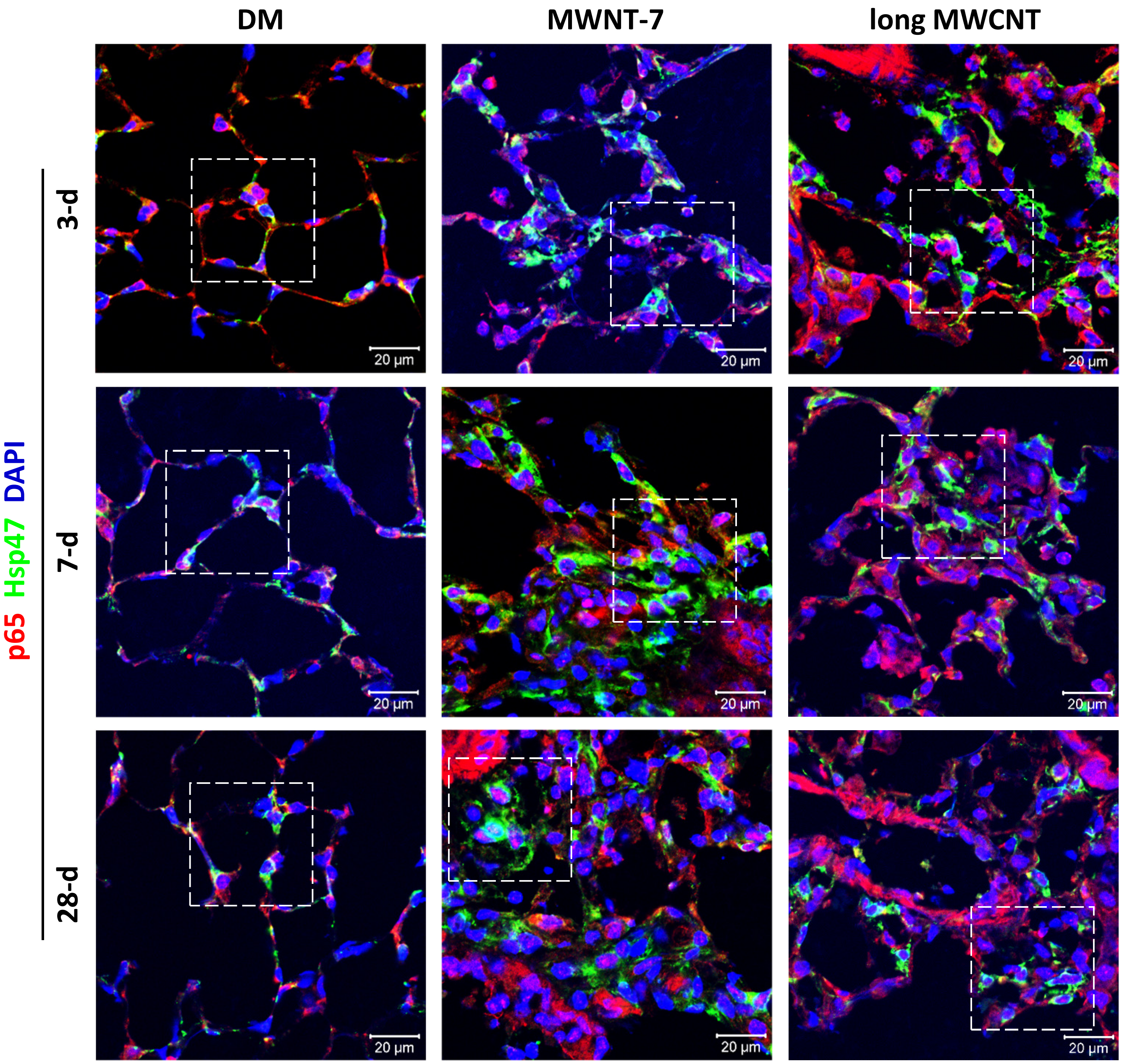

Fig. S2

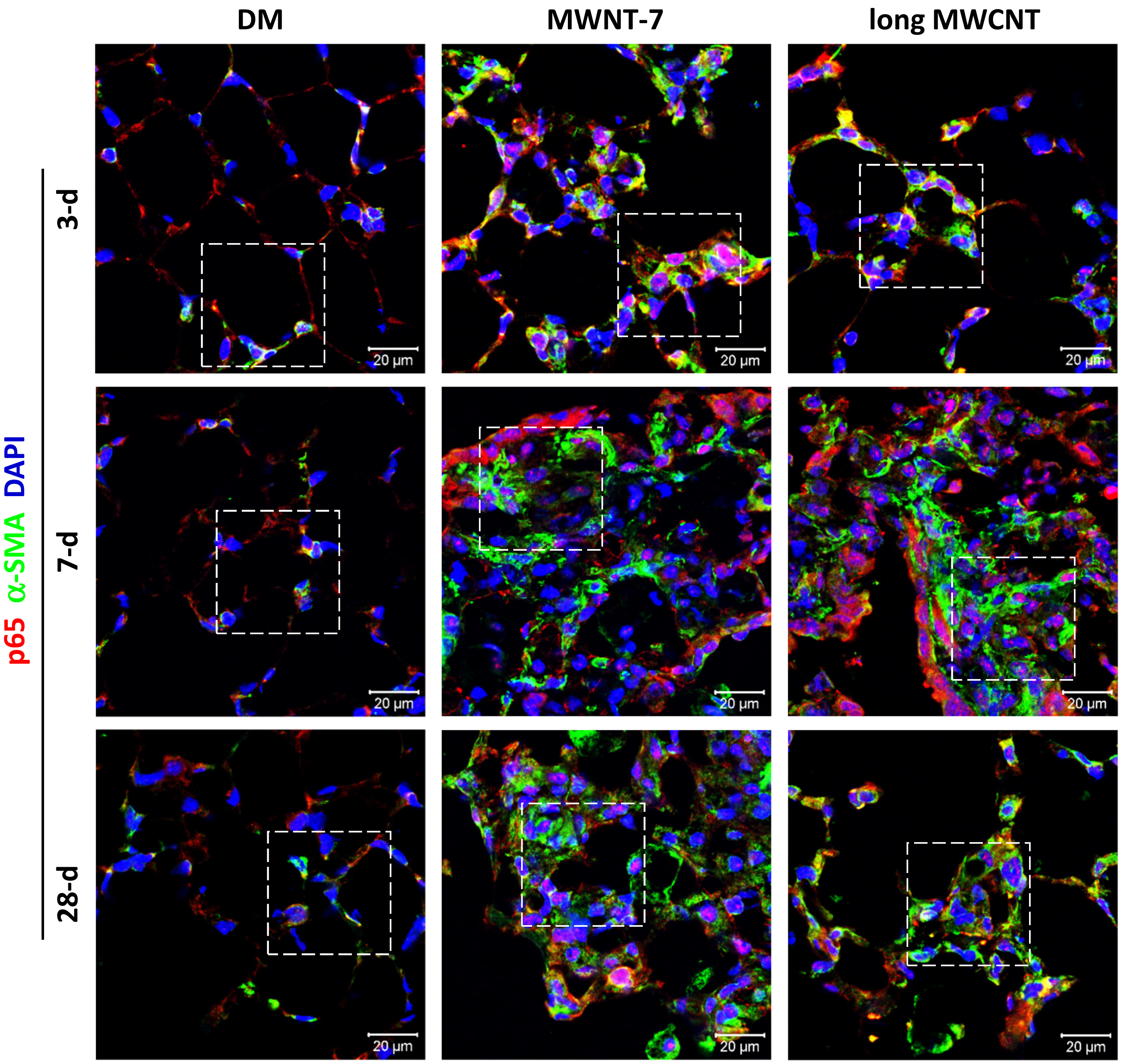

Fig. S3

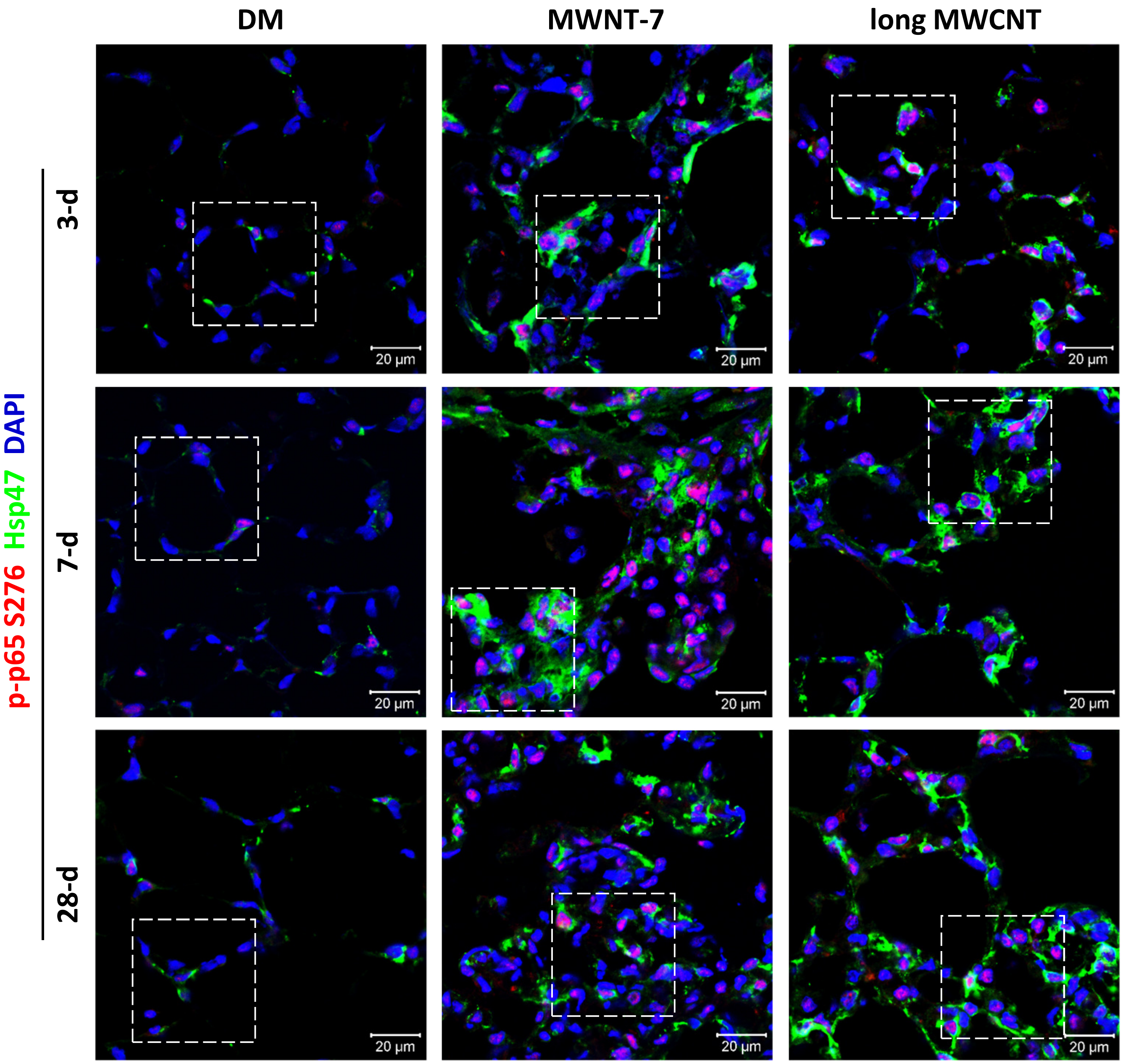

Fig. S4

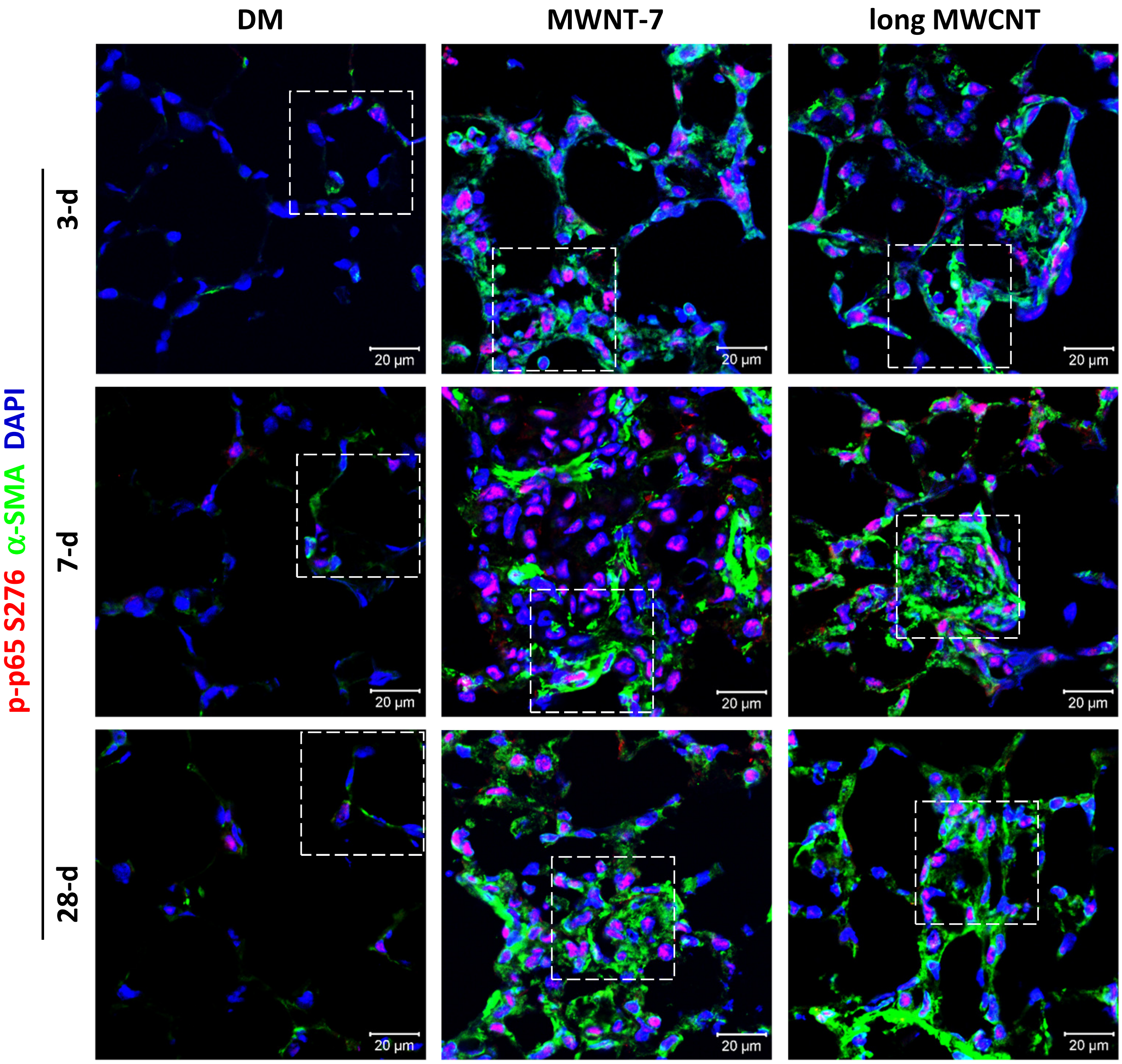

Fig. S5

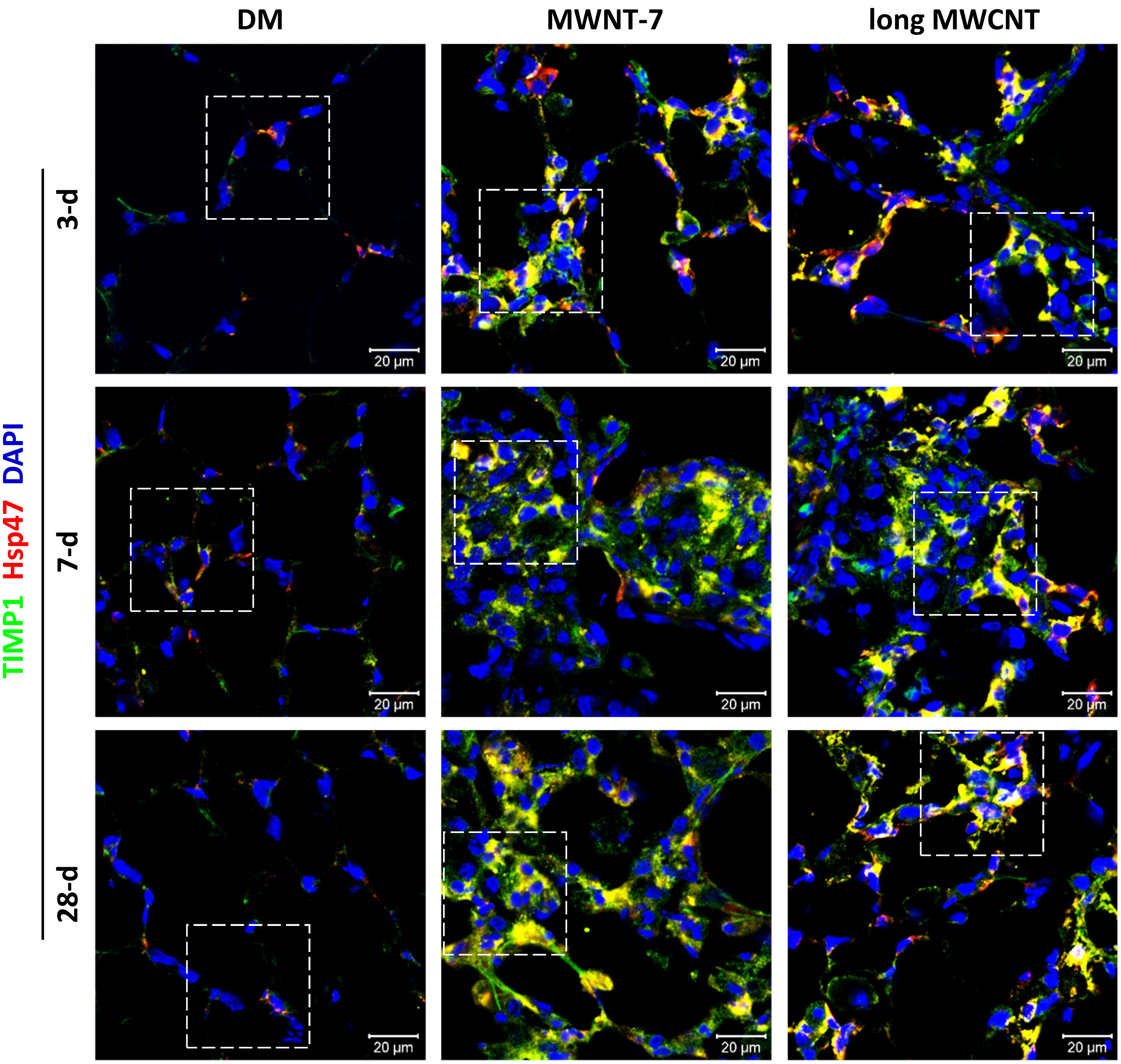

Fig. S6

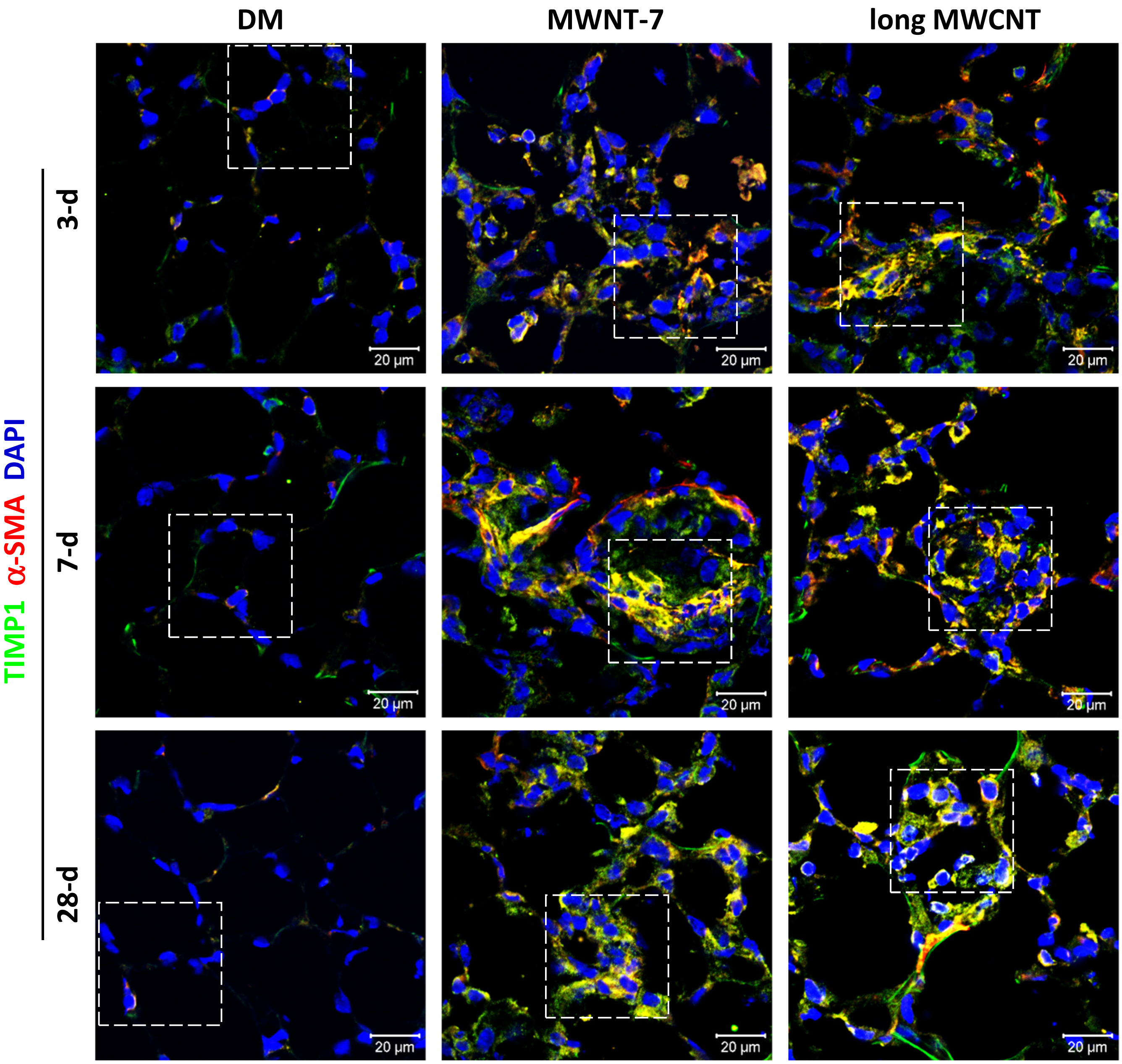

Fig. S7

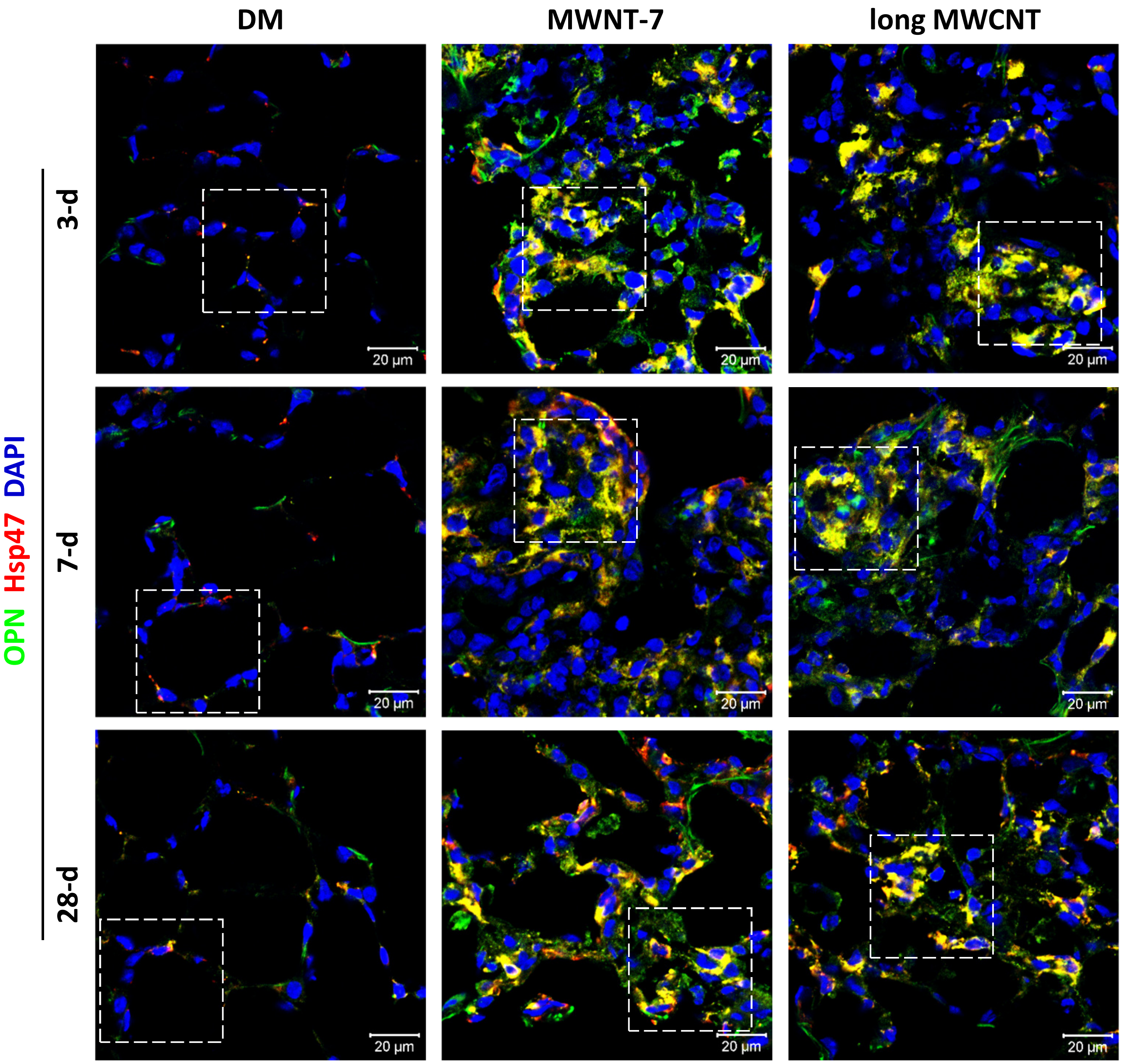

Fig. S8

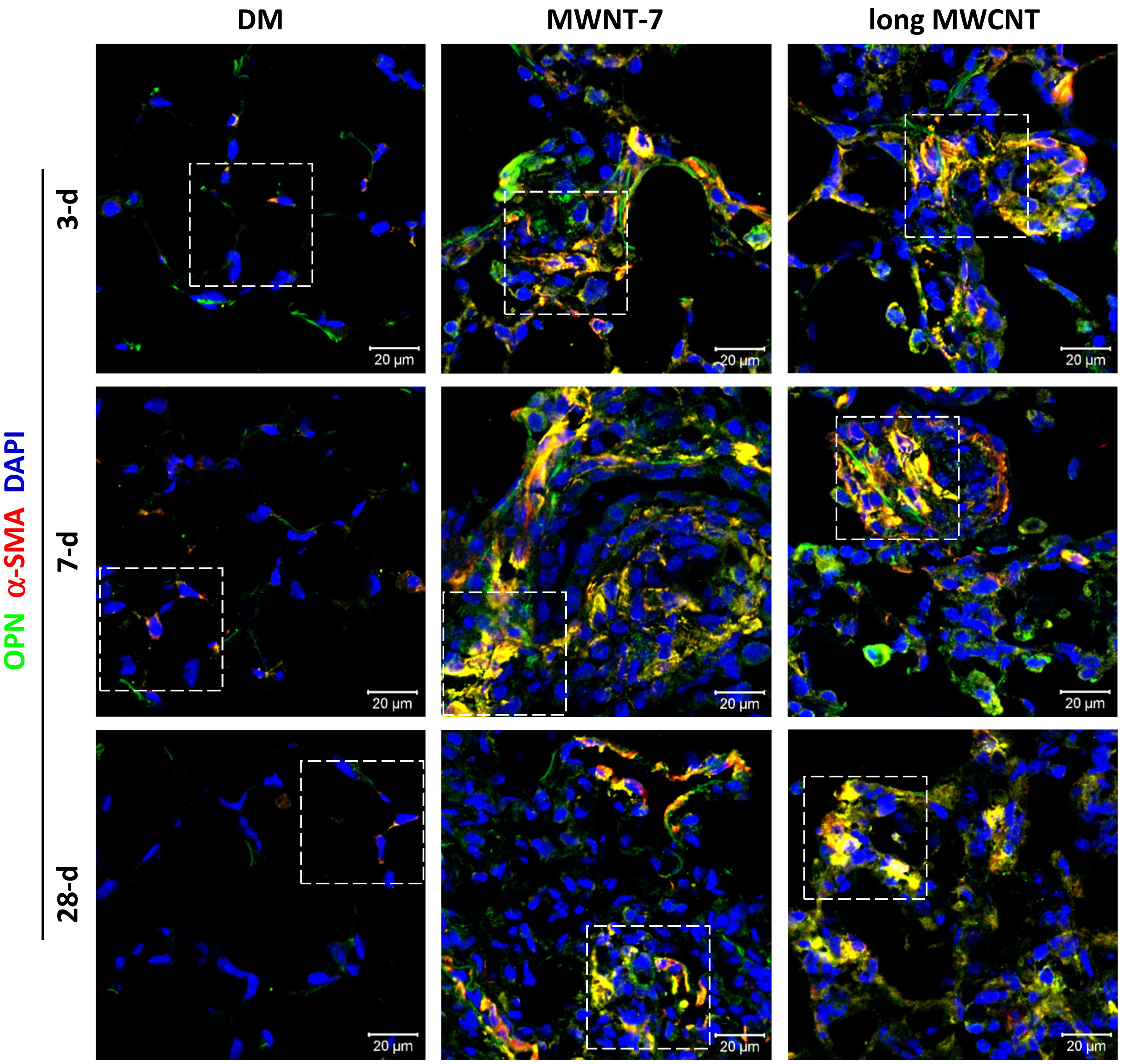

Fig. S9

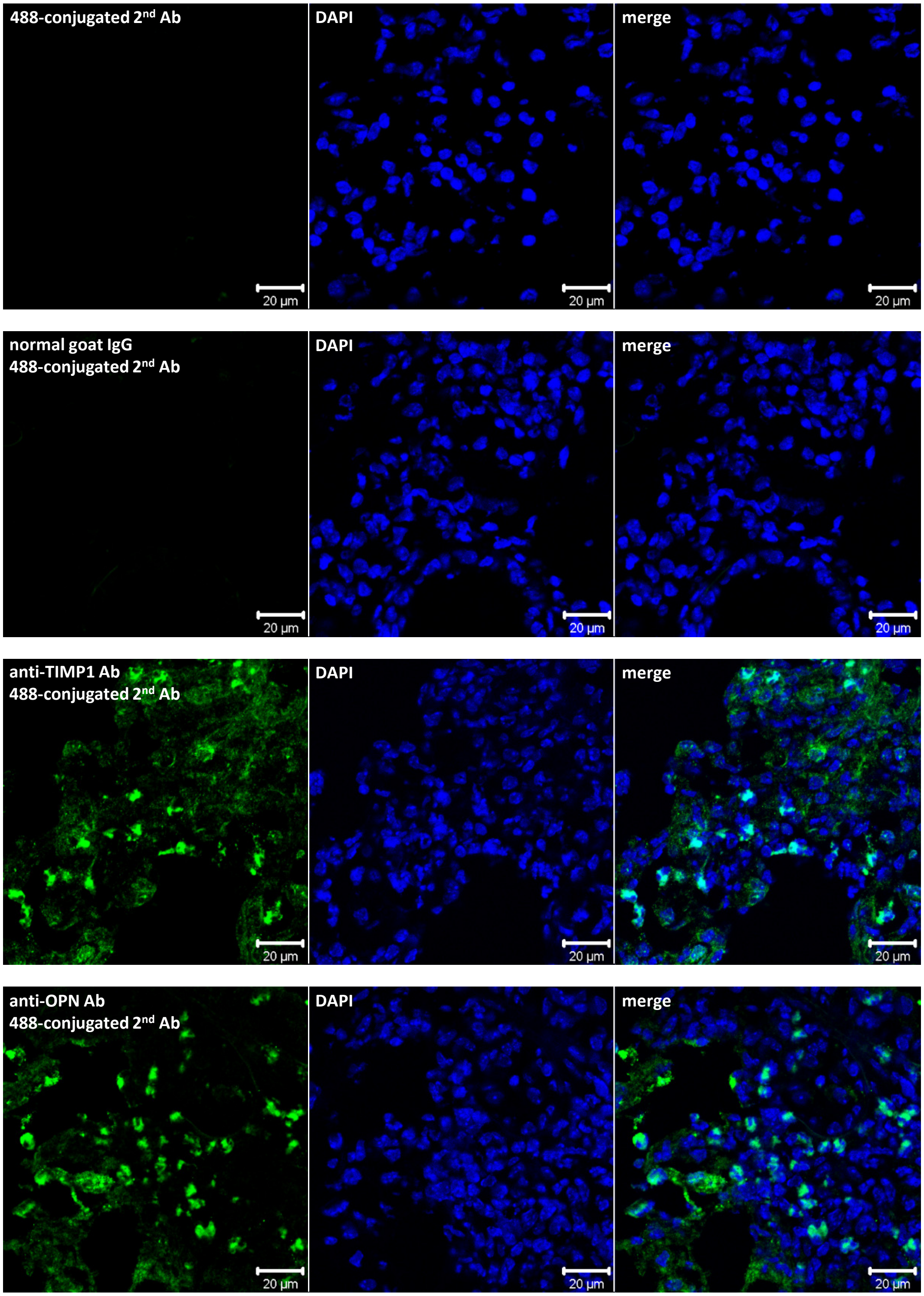
488-conjugated 2nd Ab
DAPI
merge
normal goat IgG
488-conjugated 2nd Ab
anti-TIMP1 Ab
488-conjugated 2nd Ab
anti-OPN Ab
488-conjugated 2nd Ab
20 µm
